# PReS-FINAL-2110: Tocilizumab for patients with oligoarticular juvenile idiopathic arthritis refractory to conventional therapy

**DOI:** 10.1186/1546-0096-11-S2-P122

**Published:** 2013-12-05

**Authors:** T Sato, K Nishimura, T Nozawa, T Kanetaka, M Kikuchi, R Hara, N Sakurai, K Yamazaki, Y Takeuchi, S Yokota

**Affiliations:** 1 Pediatrics, Yokohama City University School of medicine, Yokohama, Japan; 2 Pediatrics, Shiga University of Medical Schience Hospital, Shiga, Japan

## Introduction

Because most children with oligoarticular juvenile idiopathic arthritis (o-JIA) were mildly affected (Steinblocker functional class I, c.a.85%), o-JIA is tend to be thought of as a mild subtype of JIA. However, 6% of them were unable to participate in a full school program 5 years after diagnosis, and 0.5% of children with o-JIA progressed to class III or IV, severe to terminal stages. Moreover, in 20% of o-JIA patients there is a progressive increase in the number of affected joints after the first 6 months of disease (extended type). These severe patients had never gone into remission despite the conventional therapy.

## Objectives

To assess the efficacy and adverse events of tocilizumab for children with o-JIA refractory to the methotrexate (MTX) and prednisone (PSL) therapy.

## Methods

Eight patients with o-JIA refractory to MTX and PSL therapy were eligible in this study, including 7 patients with a persistent type and 1 patient with an extended type. Synovial inflammation and joint effusion were demonstrated by ultrasound and power Doppler examination as well as physical examination and other laboratory findings. Patients complicated with uveitis were excluded from the study because their arthritis had responded well to the conventional therapy. All patients were female, and the median age at analysis was 10.1 years. Both the rheumatoid factor and the anti-CCP antibody were positive in the same 4 patients. Tocilizumab (8 mg/kg) was infused every 4 weeks. Efficacy and tolerability were assessed by VAS28-CRP, matrix metalloproteinase (MMP)-3 levels, and PSL doses.

## Results

The affected joints were both knee joints (2/8), one knee joint (2/8), right knee joint and both foot joints (1/8), and both wrist joints (2/8). One patient with the extended type had arthritis primarily on knee and foot joints that extended to elbow and wrists joints within 6 months. The number of tender and swollen joints were decreased to none in 5 out of 8 patients within 6 months. Only one patient needed 12 months to become arthritis-free after TCZ initiation. The serum levels of MMP-3 in all patients were within baseline levels during the 6 months with TCZ treatment, though in 5 of them, MMP-3 levels were in the normal range at the beginning of TCZ administration. The mean doses of PSL were 7.5 mg/day before TCZ. They decreased to 3.1 mg/day after 6 months with TCZ, and then to none after 12 months. No serious adverse events were observed.

## Conclusion

TCZ was effective and well tolerated for patients with o-JIA refractory to conventional treatments.

## Disclosure of interest

None declared.

